# Messenger RNA Sequence Rather than Protein Sequence Determines the Level of Self-synthesis and Antigen Presentation of the EBV-encoded Antigen, EBNA1

**DOI:** 10.1371/journal.ppat.1003112

**Published:** 2012-12-27

**Authors:** Judy T. Tellam, Lea Lekieffre, Jie Zhong, David J. Lynn, Rajiv Khanna

**Affiliations:** 1 Tumour Immunology, Department of Immunology, Clive Berghofer Cancer Research Centre and Australian Centre for Vaccine Development, Queensland Institute of Medical Research, Herston, Queensland, Australia; 2 Animal and Bioscience Research Department, AGRIC, Teagasc, Grange, Dunsany, Co. Meath, Ireland; University of Wisconsin-Madison, United States of America

## Abstract

Unique purine-rich mRNA sequences embedded in the coding sequences of a distinct group of gammaherpesvirus maintenance proteins underlie the ability of the latently infected cell to minimize immune recognition. The Epstein-Barr virus nuclear antigen, EBNA1, a well characterized lymphocryptovirus maintenance protein has been shown to inhibit *in cis* antigen presentation, due in part to a large internal repeat domain encoding glycine and alanine residues (GAr) encoded by a purine-rich mRNA sequence. Recent studies have suggested that it is the purine-rich mRNA sequence of this repeat region rather than the encoded GAr polypeptide that directly inhibits EBNA1 self-synthesis and contributes to immune evasion. To test this hypothesis, we generated a series of EBNA1 internal repeat frameshift constructs and assessed their effects on *cis*-translation and endogenous antigen presentation. Diverse peptide sequences resulting from alternative repeat reading frames did not alleviate the translational inhibition characteristic of EBNA1 self-synthesis or the ensuing reduced surface presentation of EBNA1-specific peptide-MHC class I complexes. Human cells expressing the EBNA1 frameshift variants were also poorly recognized by antigen-specific T-cells. Furthermore, a comparative analysis of the mRNA sequences of the corresponding repeat regions of different viral maintenance homologues highlights the high degree of identity between the nucleotide sequences despite very little homology in the encoded amino acid sequences. Based on these combined observations, we propose that the *cis*-translational inhibitory effect of the EBNA1 internal repeat sequence operates mechanistically at the nucleotide level, potentially through RNA secondary structural elements, and is unlikely to be mediated through the GAr polypeptide. The demonstration that the EBNA1 repeat mRNA sequence and not the encoded protein sequence underlies immune evasion in this class of virus suggests a novel approach to therapeutic development through the use of anti-sense strategies or small molecules targeting EBNA1 mRNA structure.

## Author Summary

Viruses establishing persistent latent infections have evolved various mechanisms to avoid immune surveillance. The Epstein-Barr virus-encoded nuclear antigen, EBNA1, expressed in all EBV-associated malignancies, modulates its own protein levels at quantities sufficient to maintain viral infection but low enough so as to minimize an immune response by the infected host cell. This evasion mechanism is regulated through an internal purine-rich mRNA repeat sequence encoding glycine and alanine residues. In this study we assess the impact of the repeat's nucleotide versus peptide sequence on inhibiting EBNA1 self-synthesis and antigen presentation. We demonstrate that altered peptide sequences resulting from frameshift mutations within the repeat do not alleviate the immune-evasive function of EBNA1, suggesting that the repetitive purine-rich mRNA sequence itself is responsible for inhibiting EBNA1 synthesis and subsequent poor immunogenicity. Our comparative analysis of the mRNA sequences of the corresponding repeat regions of different gammaherpesvirus maintenance homologues to EBNA1 highlights the high degree of identity between the nucleotide sequences despite very little homology in the encoded amino acid sequences. These studies demonstrate the importance of gammaherpesvirus purine-rich mRNA repeat sequences on antigenic epitope generation and evasion from T-cell mediated immune control, suggesting novel approaches to prevention and treatment of latent infection by this class of virus.

## Introduction

Members of the viral family *Herpesviridae*, which are widely distributed throughout the animal kingdom, are characterized by their large double-stranded, linear DNA genomes. The gammaherpesviruses, one of three sub-families of *Herpesviridae*, predominantly replicate and persist in lymphoid cells with the distinguishing characteristic that they are able to establish lifelong latent infections of their hosts [1].

Gammaherpesviruses are of particular interest mainly due to the two human viruses, Epstein-Barr virus (EBV) and Kaposi's sarcoma-associated herpes virus, (KSHV) and the diseases they cause; Burkitts lymphoma, Nasopharyngeal carcinoma and Hodgkins lymphoma in the case of EBV and Kaposi's sarcoma, primary effusion lymphomas and AIDS-related lymphoproliferative disorders in the case of KSHV [2]–[4]. Latent infection of host cells by many gammaherpesviruses is dependent upon the expression of a viral maintenance protein, which ensures persistence of the viral episome within actively dividing cells, yet simultaneously evades immune surveillance [5]–[8]. Recent studies have investigated the unique properties of gammaherpesvirus maintenance proteins that allow the virus to restrict detection by host CD8^+^ cytotoxic T lymphocytes (CTLs) at crucial times during latency [1], [9]–[16].

Expression of the EBV nuclear protein 1 (EBNA1) is widespread in all forms of EBV infection, accentuating its central role in the maintenance of the viral DNA episome, a process essential for viral persistence and associated oncogenic potential [17], [18]. A wide range of studies have demonstrated that EBV latently infected B cells are able to escape immune recognition, due in part to an internal glycine–alanine repeat (GAr) domain within EBNA1, which significantly limits MHC class I-restricted presentation of EBNA1 epitopes linked *in cis* [9]–[15], [19]–[22]. An earlier report suggested that the GAr polypeptide directly interfered with the translational machinery [10]. However, more recent studies including reports from the Hoeben group, have proposed that the EBNA1 purine-rich mRNA secondary structure encoding the GAr, rather than the protein sequence, is the critical component underlying the regulation of self-synthesis and evasion of immune recognition by cytotoxic T-cells [9], [11], [12], [15], [22].

Similar to EBNA1, the latency-associated nuclear antigen 1 (LANA1) maintenance protein of the closely related KSHV virus also acts to tether the viral episome to the host genome, thereby permitting the necessary segregation of viral DNA during cell division [9], [15]. Studies have also demonstrated that LANA1 inhibits MHC class I peptide presentation *in cis* as a means of immune evasion [9], [15]. Interestingly, studies of several other members of the gammaherpesvirus family have also reported similar immune evasive properties for the maintenance proteins of these viruses [23], [24].

To define the underlying mechanism influencing the *cis*-translational inhibition responsible for minimizing the exposure of EBNA1 epitopes to immune surveillance, we have designed a series of EBNA1 expression constructs encoding alternative repeat reading frames to assess their impact on self-synthesis and antigen presentation. As well as these genetic experiments, we have also undertaken a detailed comparative analysis of the mRNA and protein sequences of the repeat regions of different gammaherpesvirus maintenance protein homologues. Based on these analyses, we conclude that the *cis*-inhibitory effect of the internal repeat sequences of gammaherpesviruses operates at the nucleotide level and is unlikely to be mediated through the direct action of the GAr polypeptide.

## Results

### Comparative analysis of the mRNA sequences encoding different gammaherpesvirus maintenance proteins

The internal GAr sequence within the EBV maintenance protein, EBNA1, has been shown to inhibit self-synthesis, which in turn significantly restricts *in cis* antigen presentation [10]–[14], [19]–[22]. To assess the functional importance of the mRNA sequence versus the protein sequence of the EBNA1 internal repeat in inhibiting self-synthesis, we compared both the mRNA and encoded protein sequences of similar internal repeat structures within the viral maintenance proteins of several gammaherpesviruses. Similar to EBNA1, these maintenance proteins are critical for the persistence of the viral genome within latently infected cells. Gammaherpesviruses have been subdivided into four genera: *Lymphocryptovirus*, *Rhadinovirus*, *Macavirus* and *Percavirus* (Table 1) [25]. Lymphocryptoviruses (LCVs) include the well-characterized EBV or *Human herpesvirus 4* [26], [27], Lymphocryptovirus of rhesus monkeys, and Herpesvirus papio of baboons [26], [28], [29]. The Rhadinoviruses include the second human gammaherpesvirus KSHV or *Human herpesvirus 8* [30], [31], Herpesvirus saimiri (HVS) [32] and Rhesus monkey rhadinovirus (RRV) [33]. The genera *Macavirus* includes the *Alcelaphine herpesvirus 1* [34] and a newly defined species [25] the *Ovine herpesvirus 2* (Table 1) [35]. The coding mRNA sequence and deduced protein sequence of the viral maintenance proteins of these gammaherpesviruses were extracted from GenBank [36].

**Table 1:** Homologies among gammaherpesvirus maintenance proteins.

Gammaherpesviruses	Viral maintenance protein homologues	Internal mRNA repeat size (bp)	Purine content of the mRNA repeats (%)	Identity of the nucleotide sequence repeat in EBNA1 with nucleotide repeats in other viral maintenance protein homologues (%)	Homology^2^ of the protein sequence repeat in EBNA1 with repeats in other viral maintenance protein homologues (%)
***Lymphocryptoviruses***					
*Human herpesvirus 4*	EBNA1	711	88.1	100	100
*Macacine herpesvirus 4*	rhEBNA1	141	78.0	70.2	46.8
*Papiine herpesvirus 1*	baEBNA1	49	80.9	75.6	55
***Rhadinoviruses***					
*Human herpesvirus 8*	LANA1 CR1^1^	1818	76.2	52	0.3
	CR2			50	0.3
	CR3			72	0.4
*Macacine herpesvirus 5*	ORF73	1554	66.5	57.1	2.1
*Saimirine herpesvirus 2*	ORF73	546	85.3	58.5	17.6
***Macaviruses***					
*Alcelaphine herpesvirus1*	ORF73 CR1	2061	96.6	56.6	29.5
	CR2			62.6	0
	CR3			74	50
*Ovine herpesvirus 2*	ORF73 CR1	984	85.7	65.8	40.8
	CR2			64	0
	CR3			68.4	44

1 CR 1–3 designates sub-regions of internal central repeats displaying varying peptide sequences.

2 Homology data for protein sequences was acquired using the Strider sequence alignment program that uses the BLOSUM62 score matrix to score pairs of aligned residues [42].

**Figure 1:**
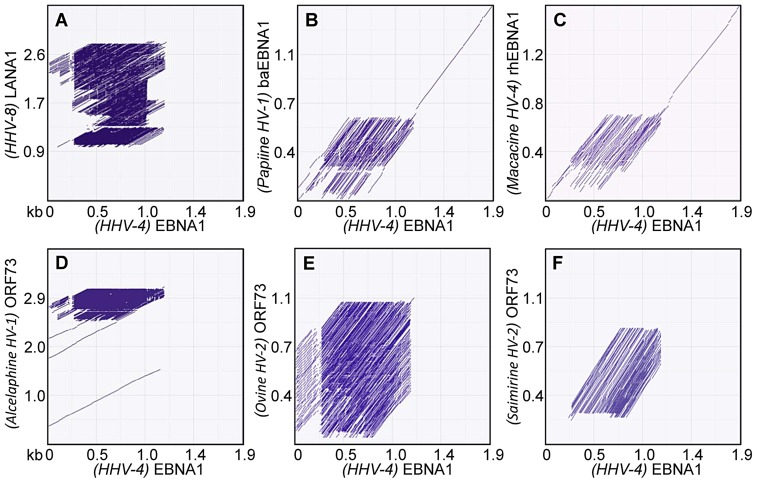
Dot-plot analyses illustrating pair wise local alignments between the EBNA1 mRNA sequence and the mRNAs for several gammaherpesvirus maintenance protein sequences. The over-all homology between sequences is shown as a straight line on the diagonal, while regions of repeats are shown as lots of lines in the same region, allowing visualization of where the repeated regions are between sequences. Panel A, Nucleotide sequence homology between *human herpesvirus-4*, *(HHV-4)* EBNA1 and *human herpesvirus-8 (HHV-8)* LANA1; Panel B, *(Papiine herpesvirus-1, HV-1)* baboon EBNA1; Panel C, *(Macacine HV-4)* rhesus EBNA1; Panel D, *(Alcelaphine HV-1)* ORF73; Panel E, *(Ovine HV-2)* ORF73, and; Panel F, *(Samirine HV-2)* ORF73. In each panel the intensity of the dot plots indicate the level of homology between the sequences being compared. Transcript sizes are shown in kb on both axes. The Genbank accessions numbers for these sequences are listed in the Materials and Methods.

The overall homology between the EBNA1 coding mRNA sequence and coding mRNAs for different gammaherpesvirus maintenance proteins was investigated by performing mRNA dot-plot pair wise sequence alignments [37] to visualize local alignments of repeated regions between the maintenance protein homologues and EBNA1 (Fig. 1). The over-all homology between sequences is shown as a straight line on the diagonal, while regions of repeats are shown as lots of lines in the same region, allowing visualization of where the repeated regions are between sequences. In each panel the intensity of the dot plots indicate the level of homology between the sequences being compared. As illustrated in Figure 1, the EBNA1 internal mRNA repeat sequence is highly identical to regions of similar repeat sequences, albeit in different positions within the coding sequences of the maintenance proteins from other gammaherpesviruses. The plot in Panel A highlights a highly repetitive homologous region between *Human Herpes virus 4* EBNA1 (280–1180 bp) and *Human Herpes virus 8* LANA1 (1000–2800 bp), while Panel B highlights a highly repetitive homologous region towards the 5′ ends of both the *Human Herpes virus 4* EBNA1 (280–1180 bp) and *Papiine Herpes virus 1* baboon EBNA1 (290–580 bp) sequences. All six viral maintenance protein mRNAs showed varying sized repeated regions that have strong homology with the internal repeat present within the EBNA1 mRNA. In Table 1 it is apparent that the identity between these purine-rich mRNA repeat sequences of EBNA1 and other viral maintenance proteins is relatively high (50–75.6%), whilst strikingly the corresponding repeat amino acid sequences showed markedly reduced identity levels and in some cases the complete absence of any similar conservation. For example, there is less than 1% homology between *Human Herpes virus 4* EBNA1 and *Human Herpes virus 8* LANA1 repeat amino acid sequences and only 2.1% homology between *Human Herpes virus 4* EBNA1 and *Macacine herpesvirus 5* Rhesus rhadinovirus ORF73 repeat amino acid sequences, despite corresponding repeat mRNA identities of 76.2% and 66.5%, respectively.

### EBNA1 gene constructs expressing diverse protein repeat sequences but near identical mRNA sequences

**Figure 2:**
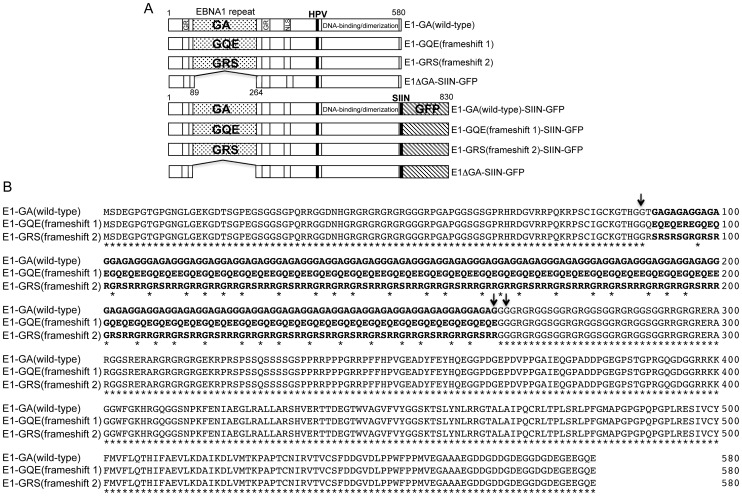
Schematic description of EBNA1 expression constructs containing identical mRNA sequences whilst encoding alternative repeat reading frames. A, The EBNA1 frameshift constructs were generated in either pcDNA3 for *in vitro* translation studies or in pEGFP-N1 for EBNA1-GFP expression and immunological studies. The overlapping DNA binding and dimerization domain, nuclear localization signal (NLS) and Glycine/Arginine (GR) repeat flanking sequences essential for genome maintenance functions are shown. The localizations of a model SIINFEKL epitope and the endogenous EBNA1 HPVGEADYFEY epitope used in the presentation assays are highlighted. B, Alignment of amino acid sequences of the EBNA1 (E1) repeat frameshift variants E1-GA(wild-type), E1-GQE(frameshift 1) and E1-GRS(frameshift 2). An asterisk indicates identical residues in all three proteins. Arrows denote nucleotide deletion positions at the start of the internal repeat and nucleotide insertion positions at the ends of the repeat in the E1-GA(wild-type) sequence to generate alternative repeat reading frames whilst maintaining wild-type EBNA1 sequence at both the N- and C-terminal domains flanking the internal repeat sequence.

Three EBNA1 expression constructs were designed comprising identical mRNA sequences whilst encoding three, alternative repeat reading frames. The constructs were used to assess the impacts of the EBNA1 repeat region mRNA and protein sequence on self-synthesis and antigen presentation. Three DNA fragments were synthesized to generate the alternative EBNA1 repeat reading frames encoding either glycine/alanine residues, referred to as E1-GA(wild-type); glycine/glutamine/glutamic acid residues, referred to as E1-GQE(frameshift 1); or glycine/arginine/serine, referred to as E1-GRS(frameshift 2). The synthesized DNA fragments were cloned into an EBNA1 expression construct lacking the internal GAr sequence (E1ΔGA/pcDNA3) to generate the EBNA1 protein sequences outlined in Figure 2. This strategy maintained the wild-type protein sequences in the regions flanking the internal repeat. As illustrated in Figure 2, a single nucleotide deletion near the start of the EBNA1 repeat sequence generated a strongly acidic (GQE) repeat domain, whilst the deletion of two nucleotides at the same position resulted in a third repeat reading frame encoding a repetitive peptide with both basic and neutral residues (GRS). The corresponding insertion of either one or two nucleotides at the end of the repeat sequence allowed the contiguous encoded C-terminal domains for these constructs to maintain wild-type EBNA1 protein sequence (Figure S1 in Text S1). Thus, the three proteins generated by these constructs were highly dissimilar in their repeat regions in terms of amino acid composition and charge.

**Figure 3:**
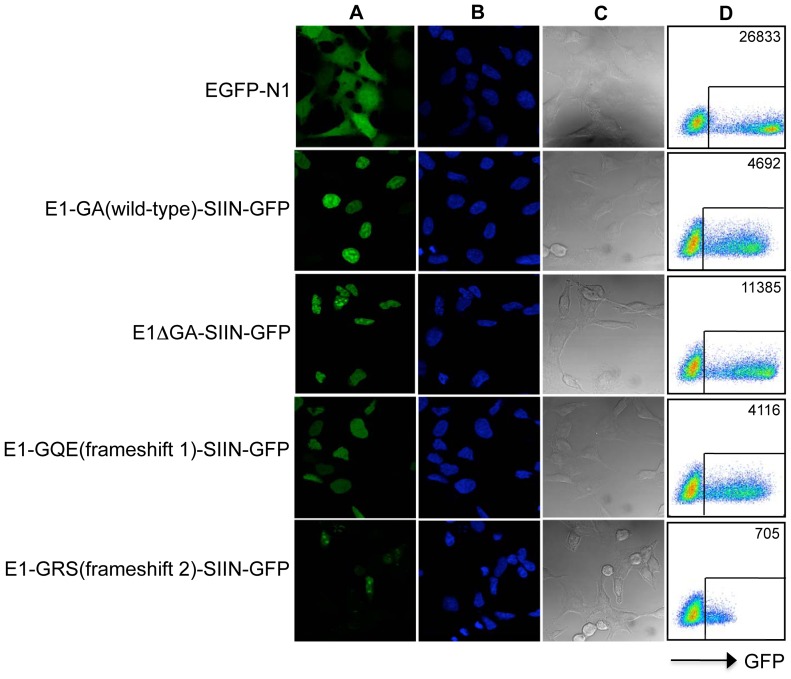
Localization and expression of EBNA1-SIIN-GFP frameshift constructs. Panel A, GFP fluorescence of EGFP-N1, E1-GA(wild-type)-SIIN-GFP, E1ΔGA-SIIN-GFP, E1-GQE(frameshift 1)-SIIN-GFP or E1-GRS(frameshift 2)-SIIN-GFP expression constructs in 293KbC2 cells. The cells were examined using a laser-scanning Bio-Rad (Hercules, CA) MRC600 confocal microscope with original magnification ×63. Panel B, DAPI staining, and; Panel C, phase contrast images of the EBNA1-SIIN-GFP transfected cells. Panel D, Flow cytometric analysis of GFP expression in 293KbC2 cells following transfection with EGFP-N1 or EBNA1-SIIN-GFP frameshift variants. The Mean Fluorescence Intensity (MFI) of the EBNA1-GFP positive cells is indicated in the top right hand corner of each plot.

For intracellular localization studies, the EBNA1 frameshift expression sequences were also sub-cloned in-frame with a sequence coding for green fluorescent protein, generating fusion proteins with GFP at the C-terminus. In addition, the H-2K^b^-restricted SIINFEKL epitope from ovalbumin, was inserted in-frame into the different EBNA1-GFP frameshift variants for endogenous processing studies thereby generating the following expression constructs E1ΔGA-SIIN-GFP, E1-GA(wild-type)-SIIN-GFP, E1-GQE(frameshift 1)-SIIN-GFP and E1-GRS(frameshift 2)-SIIN-GFP. A microscopic analysis presented in Figure 3 (panels A and B) demonstrated an unaltered nuclear staining pattern for all three EBNA1-SIIN-GFP frameshift variants as well as for E1ΔGA-SIIN-GFP, which lacks the internal repeat. Whilst constructs encoding the wild-type GA repeat or GQE repeat resulted in similar expression levels following transfection, there was reduced EBNA1-GFP expression for the construct encoding GRS repeat sequences (Fig. 3, panel A). The reduced expression of E1-GRS(frameshift 2)-SIIN-GFP transfectants was not due to differential transfection efficiencies as all three alternative reading frame constructs contain virtually identical DNA sequences which differ by only one or two nucleotides. The lower expression of E1-GRS(frameshift 2)-SIIN-GFP transfectants was consistent with decreased cell viability observed in the phase contrast microscopic analysis of the GRS transfectants (Figure 3, panel C), demonstrating a reduced percentage of GFP-expressing cells following transfection. Also of note, the GFP+ve GRS-transfectants are very low GFP expressers (MFI of 705) compared to an MFI of 4692 for the EBNA1 wild-type transfectants and 11,385 for E1ΔGA transfectants, indicating less EBNA1-GFP is being synthesized in GRS transfectants (Figure 3 panel D) and possibly resulting in a percentage of the GRS GFP-expressing cells being below the threshold level for GFP detection. Expression levels of the EBNA1-SIIN-GFP frameshift variants were also confirmed by flow cytometry (Fig. 3, panel D). Thus, the three EBNA1-GFP frameshift variants are all expressed and demonstrate similar nuclear localization.

### Alternative EBNA1 repeat peptide sequences display similar intracellular degradation kinetics and do not lead to increased EBNA1 expression

**Figure 4:**
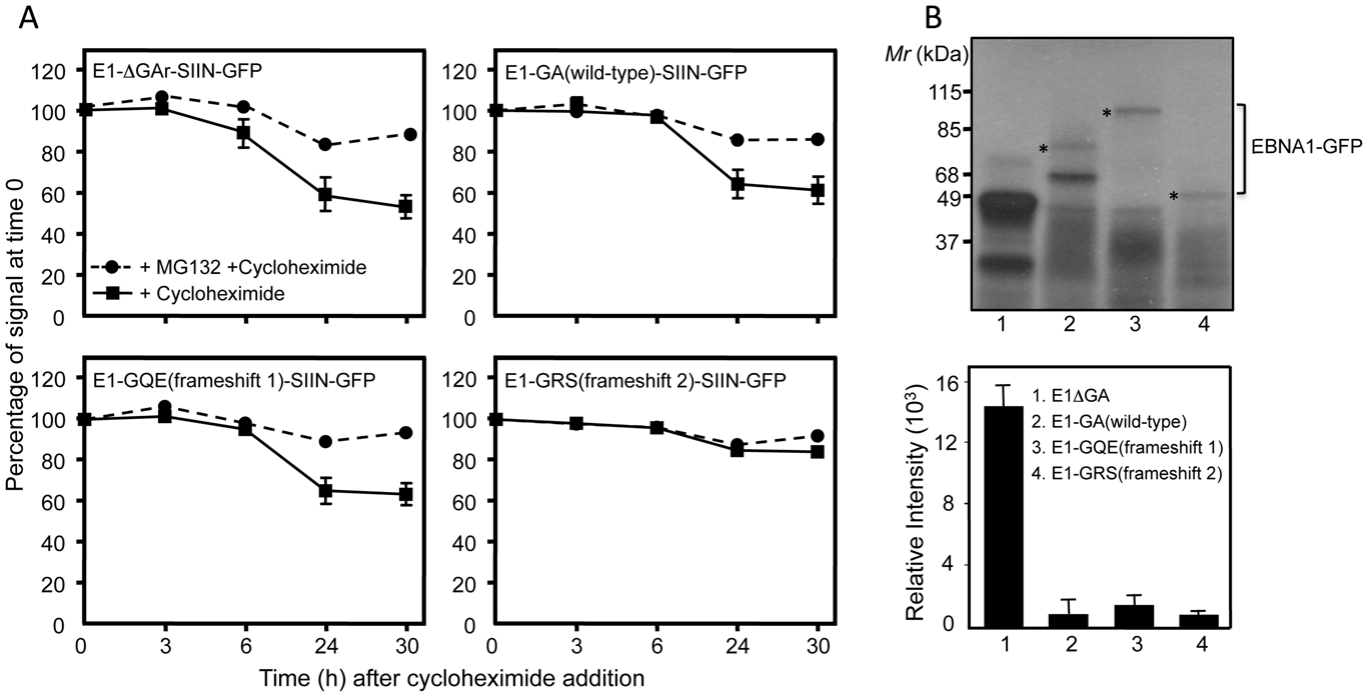
Intracellular degradation kinetics and expression of alternative EBNA1 repeat peptide sequences. A, 293KbC2 cells were transfected with the following expression constructs E1ΔGA-SIIN-GFP; E1-GA(wild-type)-SIIN-GFP; E1-GQE(frameshift 1)-SIIN-GFP or E1-GRS(frameshift 2)-SIIN-GFP in the presence or absence of the proteasome inhibitor MG132 (10 µM). At 24 h post-transfection, the cells were incubated with cycloheximide (50 µg/ml) and then monitored over a 30-h time course as described in the Materials and Methods. EBNA1-SIIN-GFP expression of the cells at each time point was monitored by flow cytometry and plotted as the relative change in the levels of EBNA1-GFP expression following the addition of cycloheximide at time point 0. B, *In vitro* translation (IVT) assay of pcDNA3 expression constructs encoding E1ΔGA (lane 1), E1-GA(wild-type) (lane 2), E1-GQE(frameshift 1) (lane 3) or E1-GRS(frameshift 2) (lane 4).The constructs were transcribed and translated *in vitro* with T7 RNA polymerase by using a coupled transcription/translation reticulocyte lysate system. ^35^S-methionine-labeled proteins were visualized by autoradiography (upper panel). An asterisk indicates the full-length translation product of each EBNA1 frameshift variant. Band intensities from the IVT assay were quantified by densitometric analysis using Imagequant software (Molecular Dynamics) and graphed to demonstrate absolute intensities (lower panel). These data are representative of three separate experiments.

To discount the possibility that altered peptide sequences due to alternative reading frames within the EBNA1 repeat domain may have changed protein stability, we determined the intracellular kinetics of degradation of the EBNA1-SIIN-GFP frameshift variants following cycloheximide treatment of 293KbC2 cells transiently transfected with E1ΔGA-SIIN-GFP, E1-GA(wild-type)-SIIN-GFP, E1-GQE(frameshift 1)-SIIN-GFP, E1-GRS(frameshift 2)-SIIN-GFP or E1ΔGA-SIIN-GFP expression constructs (in the presence or absence of 10 µM of the proteasomal inhibitor MG132) over a 30 hour time course. Both the E1-GA(wild-type)-SIIN-GFP and E1-GQE(frameshift 1)-SIIN-GFP transfectants displayed a similar pattern of degradation (Fig. 4A) which was slightly lower than that observed for E1ΔGA-SIIN-GFP transfectants. The E1-GRS(frameshift 2)-SIIN-GFP transfectants displayed a less pronounced decrease in EBNA1-SIIN-GFP expression at 24 hours (Fig. 4A). The degradation kinetics carried out in the presence of the proteasomal inhibitor MG132 demonstrate that the observed loss of EBNA1-GFP fluorescence following cycloheximide treatment is due to turnover (Figure 4A).

*In vitro* translation assays of the EBNA1/pcDNA3 frameshift variants demonstrated similarly low translational efficiencies for EBNA1 sequences encoding either GQE or GRS repeat domains as observed for the wild-type GA repeat domain (Fig. 4B). In contrast, the translational efficiency of the EBNA1 sequence lacking the internal repeat domain, E1ΔGA, is 10-fold higher (p<0.05) (Fig. 4B). The different migration rates observed for the EBNA1 frameshift variants is due to the nature of the highly repeated residues within the frameshift repeat domains leading to varying amounts of bound SDS per unit mass of protein. The strongly acidic protein, E1-GA(GQE), binds less SDS and hence migrates slower than expected. The E1-GA(GRS) protein is strongly basic and binds excess SDS causing the protein to migrate faster than expected. All of the expression constructs containing the purine-rich repeat also display premature termination products arising from the difficulty in ribosome transit through this sequence. In summary, neither of the two frameshift variants E1-GA(GQE) or E1-GA(GRS) were able to override the translational inhibition observed for the EBNA1 sequence encoding the wild-type GA repeat domain.

### Impact of repeat frameshifts on the endogenous processing of EBNA1 CD8+ T-cell epitopes

**Figure 5:**
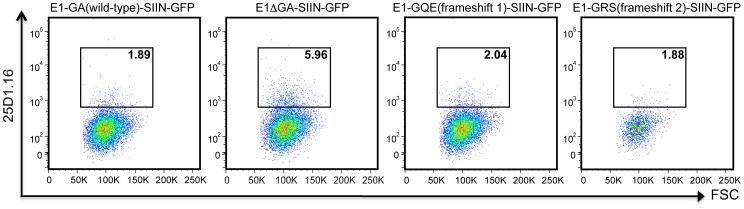
Detection of H-2K^b^–SIIN complexes on the surface of 293KbC2 cells expressing EBNA1-SIIN-GFP frameshift variants. H-2K^b^–SIIN expression was assessed by flow cytometry of 293KbC2 cells transfected with E1-GA(wild-type)-SIIN-GFP, E1ΔGA-SIIN-GFP, E1-GQE(frameshift 1)-SIIN-GFP or E1-GRS(frameshift 2)-SIIN-GFP following staining with the monoclonal antibody 25D1.16 conjugated to Allophycocyanin [38]. Values shown in each FACS plot are the percentage of GFP^+^H-2K^b^–SIIN^+^ cells as described in the Materials and Methods. These data are representative of three separate experiments.

Several experiments were undertaken to examine the impact of alternative EBNA1 repeat peptide sequences on the endogenous processing of MHC class I-restricted epitopes within EBNA1. In the first set of experiments, the endogenous loading of MHC class I molecules with a H-2K^b^-restricted epitope from ovalbumin (SIINFEKL residues 257–264) inserted at the C-terminus of the EBNA1 sequence was assessed [11]. H-2K^b^ expressing HEK293 cells were transiently transfected with either E1-GA(wild-type)-SIIN-GFP, E1ΔGA-SIIN-GFP, E1-GQE(frameshift 1)-SIIN-GFP or E1-GRS(frameshift 2)-SIIN-GFP expression constructs with transfection efficiencies of 47%, 50%, 42.8% and 8% respectively. Following transfection (24 h), cells were assessed by flow cytometry for GFP expression and surface expression of H-2K^b^-SIINFEKL complexes using a monoclonal antibody (25-D1.16) that recognizes the SIINFEKL epitope bound to H-2K^b^ molecules [38]. Flow cytometry results shown in Figure 5 demonstrate that the percentage of cells expressing surface H-2K^b^-SIINFEKL complexes was similar for all three EBNA1 repeat reading frames and ranged from 1.9%–2.0%. In contrast, a 4–4.3-fold increase in the surface expression of H-2K^b^-SIINFEKL complexes was observed for transfectants expressing EBNA1-GFP lacking the GAr domain (E1ΔGA-SIIN-GFP), indicating that all three repeat reading frames inhibited the endogenous processing of MHC class I-restricted epitopes within EBNA1 to a similar extent (Fig. 5). Transfection of a control parent plasmid without SIINFEKL provided a baseline (0.67% cells expressing surface H-2K^b^-SIINFEKL complexes) above which an increase in fluorescence would indicate specific surface expression of H-2K^b^-SIINFEKL complexes.

**Figure 6:**
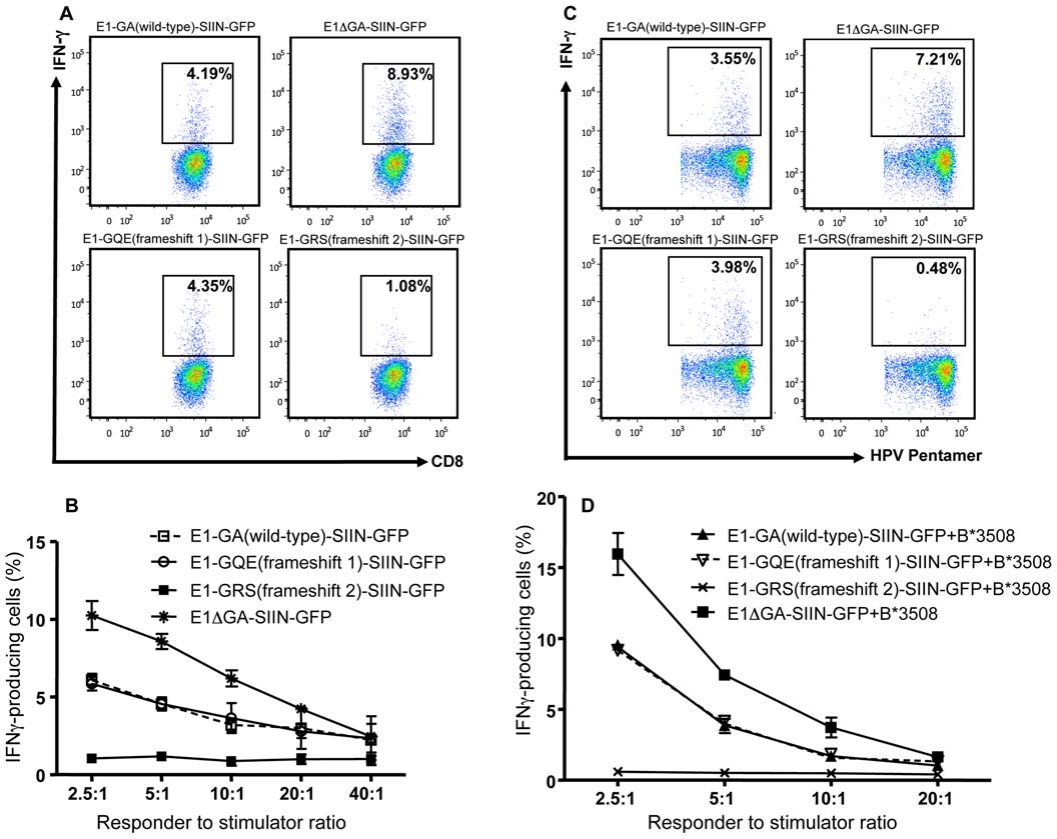
T-cell recognition of 293KbC2 cells transfected with EBNA1-SIIN-GFP frameshift variants. Panels, A and B: 293kbC2 cells expressing EBNA1-SIIN-GFP encoding either alternative repeat peptide sequences or no repeat were exposed to SIIN-specific OT-1 T-cells and then incubated for 3 hours in the presence of brefeldin A at a responder to stimulator ratio of 5∶1 (panel A) and ratios of 2.5∶1–40∶1 (panel B). Following incubation, IFN-γ production by OT-1 T-cells was determined by intracellular cytokine staining. The top right hand corner of the FACS plot in panel A indicates the percentage of OT-1-specific CD8^+^ lymphocytes producing IFN-γ. Panels, C and D: EBV-negative DG75 cells were co-transfected with EBNA1-GFP expression constructs encoding alternative repeat peptide sequences or no repeat and a HLA B*3508-GFP expression vector. The transfected cells were exposed to HPV-specific T-cells and incubated in the presence of brefeldin A overnight at a responder to stimulator ratio of 5∶1 (panel C) and ratios of 2.5∶1–20∶1 (panel D). Following incubation, IFN-γ production by HPV-specific T-cells was determined by intracellular cytokine staining and shown in the top right hand corner of the FACS plot in panel C as the percentage of HPV-specific CD8^+^ lymphocytes producing IFN-γ. These data are representative of three separate experiments.

In the next set of experiments, the influence of EBNA1 repeat frameshifts on the T-cell recognition of a H-2K^b^-restricted SIINFEKL epitope encoded within EBNA1 was evaluated. H-2K^b^ expressing HEK293 cells were transiently transfected with either E1-GA(wild-type)-SIIN-GFP, E1ΔGA-SIIN-GFP, E1-GQE(frameshift 1)-SIIN-GFP or E1-GRS(frameshift 2)-SIIN-GFP expression constructs with transfection efficiencies of 45.5%, 49%, 43% and 9.8%, respectively. Twenty-four hours post-transfection, cells were incubated with CD8^+^ T-cells specific for the SIINFEKL epitope and stimulation was assessed by intracellular cytokine staining assays (ICS). Data presented in Figure 6 (panels A and B) show that both E1-GA(wild-type)-SIIN-GFP and E1-GQE(frameshift 1)-SIIN-GFP transfectants stimulated a similar number of IFN-γ producing SIINFEKL-specific T-cells (4.2% and 4.4%, respectively). Transfectants expressing E1-GRS(frameshift 2)-SIIN-GFP stimulated 4-fold less IFN-γ producing SIINFEKL-specific T-cells (1.1%) than transfectants expressing GA(wild-type)-SIIN-GFP or GQE(frameshift 1)-SIIN-GFP, consistent with reduced EBNA1 expression levels following transfection of EBNA1-GFP constructs expressing repeat sequences encoding GRS residues. Cells expressing EBNA1-GFP lacking the GAr domain (E1ΔGA-SIIN-GFP) stimulated 2–2.1-fold more IFN-γ producing SIINFEKL-specific T-cells (8.9%) compared to GA(wild-type) (4.2%) or GQE(frameshift 1) (4.4%) repeat domains (Fig. 6, panels A and B).

The endogenous processing of a second CD8+ T-cell epitope, this time encoded within EBNA1 (HLA B*3508-restricted, HPVGEADYFEY residues 407–417) was similarly assessed. EBV-negative DG75 B-cells were transiently co-transfected with E1ΔGA-SIIN-GFP or EBNA1-GFP expression vectors encoding alternative EBNA1 repeat peptide sequences; E1-GA(wild-type)-SIIN-GFP, E1-GQE(frameshift 1)-SIIN-GFP, E1-GRS(frameshift 2)-SIIN-GFP and a HLA B*3508-GFP expression construct. Transfection efficiencies were similar for all constructs ranging from 63.2%–68.8%. Co-transfection with the HLA B*3508-GFP expression construct allowed evaluation of endogenous processing of EBNA1 using HPV-specific T-cell clones. At twenty-four hours post-transfection, cells were incubated with CD8+ T-cells specific for HPV epitopes and stimulation assessed using ICS. Data presented in Figure 6 (panels C and D) demonstrate that cells expressing E1-GQE(frameshift 1)-SIIN-GFP or E1-GRS(frameshift 2)-SIIN-GFP showed no increased stimulation of IFN-γ producing HPV-specific T-cells compared to cells expressing E1-GA(wild-type)-SIIN-GFP. This result was identical to our earlier endogenous processing and presentation data with the SIINFEKL epitope. Collectively, the endogenous processing results demonstrate that the poor immunogenicity of EBNA1 is unlikely to be due to the alanine/glycine repetitive peptide sequence within the EBNA1 repeat since alternative repeat peptide sequences also failed to enhance the presentation of MHC class I-restricted epitopes within EBNA1 to the level observed for cells expressing E1ΔGA-SIIN-GFP, where the GAr had been deleted.

### Impact of the *Ateline herpesvirus 3* ORF73 repeat mRNA on EBNA1 expression and T-cell recognition

**Figure 7:**
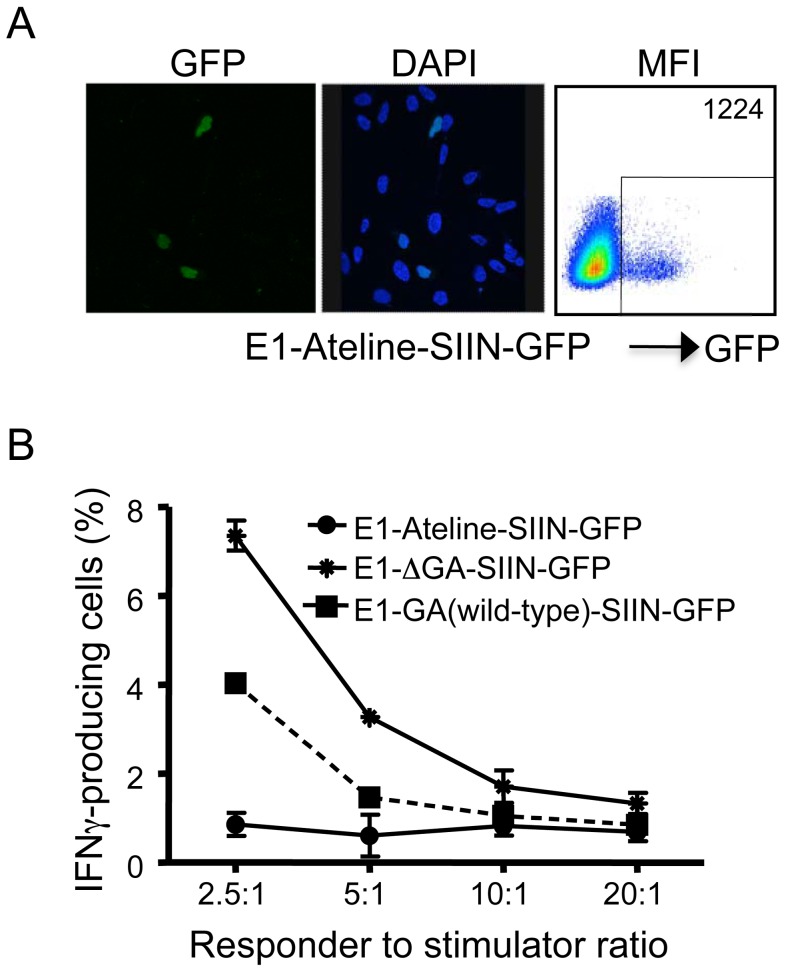
Localization, expression and T-cell recognition of 293KbC2 cells transfected with an EBNA1-Ateline-SIIN-GFP expression construct. A, GFP fluorescence, DAPI staining and Flow cytometric analysis of GFP expression in 293KbC2 cells following transfection with an EBNA1-Ateline-SIIN-GFP expression construct. The cells were examined using a laser-scanning Bio-Rad (Hercules, CA) MRC600 confocal microscope with original magnification ×63. The Mean Fluorescence Intensity (MFI) of the EBNA1-GFP positive cells is indicated in the top right hand corner of the FACS plot. B, T-cell recognition of 293KbC2 cells transfected with EBNA1-SIIN-GFP variants. 293kbC2 cells expressing EBNA1-SIIN-GFP encoding either wildtype or Ateline repeat peptide sequences or no repeat were exposed to SIIN-specific OT-1 T-cells and incubated for 3 hours in the presence of brefeldin A at responder to stimulator ratios of 2.5∶1–20∶1. Following incubation, IFN-γ production by OT-1 T-cells was determined by intracellular cytokine staining. These data are representative of three separate experiments.

To assess the impact of a purine-rich repeat sequence from another gammaherpesvirus maintenance protein on both EBNA1 expression and T-cell recognition of a SIINFEKL epitope fused to EBNA1, a 519 nucleotide repeat sequence from the *Ateline herpesvirus 3* ORF73 was synthesized and cloned into the E1ΔGA-SIIN-GFP expression vector to generate (E1-Ateline-SIIN-GFP). The resulting EBNA1-Ateline expression vectors encoded a 173 amino acid acidic repeat domain of predominantly glycine and aspartic acid (GD) residues (Figure S2 in Text S1). A microscopic analysis presented in Figure 7A demonstrated the expected nuclear staining pattern for the E1-Ateline-SIIN-GFP variant, similar to the EBNA1 wild-type and frameshift variants. Following transfection of the E1-Ateline-SIIN-GFP expression construct, we observed a notably reduced EBNA1-SIIN-GFP expression level, as evidenced by an MFI of 1224 (Fig. 7A), which was 9 fold lower than the MFI for E1ΔGA-SIIN-GFP and 3.5 fold lower than the MFI observed for E1-GA(wild-type)-SIIN-GFP (Fig. 3 panel D).

Next, the influence of the *Ateline herpesvirus 3* repeat sequence in the context of EBNA1 on T-cell recognition of the H-2K^b^-restricted SIINFEKL epitope fused to EBNA1 was also evaluated. H-2K^b^ expressing HEK293 cells were transiently transfected with either E1-Ateline-SIIN-GFP, E1ΔGA-SIIN-GFP or E1-GA(wild-type)-SIIN-GFP expression constructs. Twenty-four hours post-transfection, cells were incubated with CD8^+^ T-cells specific for the SIINFEKL epitope and stimulation assessed by intracellular cytokine staining assays. Data presented in Figure 7B demonstrate that E1-Ateline-SIIN-GFP transfectants stimulated only 1.2% of IFN-γ producing SIINFEKL-specific T-cells compared to 4.04% for E1-GA(wild-type)-SIIN-GFP transfectants and 7.59% for E1ΔGA-SIIN-GFP transfectants, at a responder∶stimulator ratio of 2.5∶1. This result is consistent with the reduced EBNA1-SIIN-GFP expression levels observed for E1-Ateline-SIIN-GFP transfectants in Figure 7A and demonstrates that the purine-rich mRNA repeat of the *Ateline herpesvirus 3* ORF73 is able to inhibit both protein expression and T-cell recognition.

## Discussion

Viruses that establish chronic latent infections of host cells have evolved numerous mechanisms to evade the host immune system. One such example is the Epstein-Barr virus nuclear antigen 1, EBNA1, which is responsible for maintenance of the viral episome within latently infected B cells. The synthesis of EBNA1 is tightly regulated to achieve levels sufficient to maintain viral infection, but low enough so as to minimize EBNA1's exposure to EBNA1-specific T-cells.

The regulated inhibition of EBNA1 synthesis has been shown to occur *in cis* as a result of an internal purine-rich repetitive mRNA sequence that dramatically reduces the rate of EBNA1 protein synthesis [12]. Removal of the repeat sequence leads to increased EBNA1 synthesis and enhanced recognition of MHC class I-restricted epitopes within EBNA1. The current studies demonstrate that this regulated inhibition of EBNA1 synthesis and the resultant restriction of antigen presentation and host immune recognition is independent of alternative repeat protein sequences embedded in EBNA1 mRNA. Combined microscopic analyses, *in vitro* translation assays and intracellular cytokine presentation experiments, investigating frameshift changes within the EBNA1 internal repeat demonstrate that altered peptide sequences within the repeat do not override the repeat's *cis*-inhibitory effect on EBNA1 translation and antigen presentation. The results show that the repetitive purine-rich mRNA sequence itself is responsible for the inhibition of EBNA1 protein synthesis and subsequent poor immunogenicity. When taken together with other studies [12], [22], these results suggest that an unusual RNA secondary structure within the repeat region may interfere with translation of the EBNA1 mRNA by inhibiting ribosome transit through the purine-rich sequence, thereby leading to a reduction in the levels of EBNA1 such that the infected cell evades the normal host immune surveillance mechanisms.

Comparison of the mRNA sequences of related viruses encoding corresponding proteins responsible for maintenance of latent infections reveals the presence of highly homologous purine-rich repetitive sequences interspersed within the functional coding regions of these proteins. Although highly conserved in mRNA sequence, these repeat regions encode very different peptide sequences in the different viruses. Moreover, substituting the native EBNA1 mRNA repeat sequence with the purine-rich mRNA repeat sequence from the related viral maintenance protein *Ateline herpesvirus 3* ORF73 demonstrated that the mRNA repeat of the *Ateline herpesvirus 3* ORF73 is able to inhibit both EBNA1-GFP expression and T-cell recognition. These observations strongly support the conclusion that the purine-rich mRNA sequence, rather than its encoded protein sequence, is responsible for the reduced expression of these viral mRNAs. The immune suppressive effects of these mRNA repeat sequences on antigenic epitope generation may represent a more general immune evasive strategy as hundreds of eukaryotic viral mRNAs have evolved with a purine bias [39].

The loss of conservation of protein sequence in the face of evolutionary conservation of the purine-rich mRNA sequence needed for translational repression and avoidance of immune surveillance may be the result of the tendency for “translational recoding” or frame-shifting that has been shown to be induced by G-rich mRNA sequences [40]. Although the overall purine-rich mRNA repeat sequence regions are strongly conserved, such an evolutionary mechanism would lead to random frame shifting and different repeat protein sequences. This suggests that the repeat sequences are subject to strong purifying selection acting at the level of the nucleotide sequence and not the protein sequence. Separate from the repeat region in EBNA1, there is a nuclear localization signal, two short domains flanking the internal GAr involved in binding to host cell chromosomes and also an overlapping DNA-binding and dimerization domain required for EBNA1 dimerization and binding to the OriP region of the viral genome, [8] [41]. Therefore, it is likely that the design of this protein serves two primary functions – viral genome maintenance and immune evasion, with the latter involving translational repression mediated by the repeat region mRNA sequence.

The identification of mRNA repeats which inhibit EBNA1 translational efficiency and endogenous antigen presentation suggests a novel approach to potential new therapeutic interventions involving the use of specific “antisense” therapeutics aimed at the putative structures in the purine-rich mRNA sequence. Such strategies would increase the amount of EBNA1 protein in latently infected cells, thus facilitating normal immune recognition and thereby elimination of the virus by the immune system.

## Materials and Methods

### Ethics statement

The Queensland Institute of Medical Research Ethics Committee approved all experiments (P353). All patients provided written informed consent for the collection of blood samples and subsequent analysis.

### Cell lines

The EBV negative cell line, DG75 was maintained in RPMI 1640 supplemented with 2 mM L-glutamine, 100 IU/ml penicillin, and 100 µg/ml streptomycin plus 10% FCS (referred to as Growth Medium) and used as targets for T-cell assays. HEK293 cells stably expressing the mouse class I allele H-2K^b^ (293KbC2) were maintained in DMEM supplemented with 5.56 mM D-glucose, 4 mM L-glutamine, 1 mM sodium pyruvate, 100 IU/ml penicillin and 100 µg/ml streptomycin plus 10% foetal calf serum (referred to as DMEM/10FCS) and were used for EBNA1 localization studies, intracellular degradation analysis and CTL assays.

### Pair wise sequence alignments

Comparisons between the coding sequence mRNA of EBNA1 and a number of other gammaherpesvirus maintenance proteins were performed using a pair wise sequence alignment visualized as dot plots. The alignments were performed using zPicture, which is a dynamic alignment and visualization tool based on the BLASTZ alignment program [37]. The Genbank accession numbers for the viral mRNAs were: *(HHV-4)* EBNA1 (NC_007605); *(HHV-8)* Lana1 (U75698.1); (*Papiine HV-1)* baboon EBNA1 (HPU23857); *(Macacine HV-4)* rhesus EBNA1 (NC_006146.1); *(Alcelaphine HV-1)* ORF73 (AF005370.1); *(Ovine HV-2)* ORF73 (AY839756.1) and *(Saimirine HV-2)* ORF73 (NC_001350.1).

### Generation of EBNA1 expression constructs

An EBNA1 expression construct encoding native GAr sequence was generated by synthesizing a 615 nucleotide DNA fragment corresponding to EBNA1 nucleotides 209–814 and incorporating a 3′ *Cla*1 site (DNA 2.0, Menlo Park CA). This DNA fragment was cloned into the *Bspe*1 and a mutagenized *Cla*1 site (position 250) of a previously generated E1ΔGA/pcDNA3 expression vector [12] to generate the expression construct E1-GA(wild-type) corresponding to native EBNA1 sequence encoding a 175 amino acid (aa) glycine/alanine repeated peptide sequence. An alternative EBNA1 frameshift expression construct was generated by altering the reading frame of the internal EBNA1 repeat sequence to encode a glycine/glutamic acid/glutamine (GQE) repeated peptide sequence (175 aa). This was achieved by synthesizing a second DNA fragment similar to that described above but with a single (A) nucleotide deletion at position 56 within the synthesized DNA fragment (corresponding to EBNA1 nucleotide position 264) to generate the expression construct E1-GQE(frameshift 1). Likewise, a second EBNA1 frameshift expression construct was generated to encode a glycine-arginine-serine (GRS) repeated peptide sequence (175 aa) by synthesizing a third DNA fragment (again similar to the first DNA fragment described above) but with two (A) nucleotides deleted at positions 56–57 within the synthesized DNA fragment (corresponding to EBNA1 nucleotide positions 264–265) to generate the expression construct E1-GRS(frameshift 2). To maintain the wildtype EBNA1 reading frame immediately following the internal repeat, either a single (G) nucleotide was inserted at EBNA1 nucleotide position 809 by mutagenesis in the E1-GQE(frameshift 1) construct or two nucleotides (AG) were inserted at EBNA1 nucleotide positions 813–814 in the E1-GRS(frameshift 2) construct. The DNA sequences of all three frameshift expression constructs did not encode stop codons as verified by DNA sequencing. The three EBNA1 frameshift DNA sequences, E1-GA(wildtype), E1-GQE(frameshift 1) and E1-GRS(frameshift 2) in addition to E1ΔGA were also sub-cloned in-frame with a sequence coding for green fluorescent protein (pEGFP-N1, CLONTECH, Palo Alto, CA) to generate E1-GA(wild-type)-GFP, E1-GQE(frameshift 1)-GFP, E1-GRS(frameshift 2)-GFP and E1ΔGA-GFP. For the assessment of endogenous loading of MHC class I molecules, a H-2K^b^-restricted epitope from ovalbumin, (Ser–Ile–Ile–Asn–Phe–Glu–Lys–Leu, residues 257–264), referred to as SIINFEKL [38] was inserted in-frame into all three EBNA1-GFP frameshift expression constructs as well as into the E1ΔGA-GFP expression construct between the 3′ end of the EBNA1 sequence and the start of the GFP sequence to generate E1-GA(wild-type)-SIIN-GFP, E1-GQE(frameshift 1)-SIIN-GFP, E1-GRS(frameshift 2)-SIIN-GFP and E1ΔGA-SIIN-GFP. Endogenous processing and surface presentation of EBNA1 was also assessed using a second epitope encoded within the EBNA1 sequence and restricted through HLA B*3508, HPVGEADYFEY (His–Pro–Val–Gly–Glu–Ala–Asp–Tyr–Phe–Glu–Tyr, residues 407–417) and referred to as HPV.

### Intracellular proteasomal inhibition and degradation studies

HEK293KbC2 cells (2×10^5^) were transiently transfected with 0.4 µg of the expression constructs E1ΔGA-SIIN-GFP, E1-GA(wild-type)-SIIN-GFP, E1-GQE(frameshift 1)-SIIN-GFP or E1-GRS(frameshift 2)-SIIN-GFP in the presence or absence of the proteasomal inhibitor MG132 (10 µM; Merck Biosciences) using Effectene (QIAGEN, Hilden, Germany) according to the manufacturer's instructions. At 24 hours post-transfection, cycloheximide (50 µg/ml) was added to each sample well. Equal aliquots of cells were trypsinized, washed and processed to measure EBNA1-GFP expression by flow cytometry at time points 0 h, 3 h, 6 h, 24 h and 30 h.

### *In vitro* translation assays

EBNA1-pcDNA3 frameshift expression constructs E1-GA(wild-type), E1-GQE(frameshift 1) and E1-GRS(frameshift 2); E1ΔGA and E1-Ateline were transcribed and translated *in vitro* with T7 RNA polymerase using a coupled transcription/translation reticulocyte lysate system (Promega, Madison WI) supplemented with 10 µCi ^35^[S]-methionine (Perkin-Elmer Pty Ltd., Boston, MA.). Lysates were subjected to SDS-PAGE followed by autoradiography and band intensities were quantified by densitometric analysis using Imagequant software (Molecular Dynamics).

### Detection of cell surface K^b^-SIINFEKL

293KbC2 cells (2×10^5^), which stably express H-2K^b^ [38], were transfected with 0.4 µg of the EBNA1-SIIN-GFP frameshift expression constructs using Effectene. A separate transfection of the parent construct without SIINFEKL was also performed to provide a negative control. Cells were harvested after an overnight transfection and stained with mAb 25D1.16 [38] conjugated to Allophycocyanin (Molecular Probes, Invitrogen) for 30 min at 4°C. Cells were washed and analyzed by flow cytometry on a FACSCanto II (BD Biosciences) for GFP expression and 25D1.16 binding.

### Intracellular cytokine staining

HEK293KbC2 cells (2×10^5^) transiently transfected with EBNA1-SIIN-GFP frameshift expression constructs (24 h) were incubated with ovalbumin-specific T-cells (OT-1) for 3 hours at 37°C at responder to stimulator ratios of 2.5∶1, 5∶1, 10∶1 and 20∶1 and 40∶1 in DMEM/10FCS medium supplemented with Brefeldin A (BD Pharmingen, San Diego, USA). Cells were washed and incubated with Allophycocyanin (APC)-conjugated anti-CD3 and PerCP-conjugated anti-CD8 for 30 min, rewashed, then fixed and permeabilized with cytofix/cytoperm (BD Pharmingen) at 4°C for 20 minutes. Cells were washed in perm/wash (BD Pharmingen), incubated with PE-conjugated anti-IFN-γ (BD Pharmingen) at 4°C for 30 mins, rewashed and analyzed for IFN-γ production by OT-1 T-cells by flow cytometry on a FACSCanto II. DG75 cells (5×10^6^) co-transfected (24 h) with 1.2 µg of EBNA1-SIIN-GFP frameshift expression constructs E1-GA(wild-type)-SIIN-GFP, E1-GQE(frameshift 1)-SIIN-GFP, E1-GRS(frameshift 2)-SIIN-GFP or E1ΔGA-SIIN-GFP and 0.8 µg of a HLA B*3508-GFP expression construct using the Amaxa Cell Line Nucleofector Kit V (Lonza, Cologne, Germany) were incubated with HPV-specific T-cells overnight (37°C) at responder to stimulator ratios of 2.5∶1, 5∶1, 10∶1 and 20∶1 in Growth Medium supplemented with Brefeldin A. IFN-γ production by HPV-specific T-cells was determined by intracellular cytokine staining as described above with FITC-conjugated anti-CD4, PerCP-conjugated anti-CD8, APC labeled B*3508 HPV Pentamer (ProImmune, Oxford, UK) and PE-conjugated anti- IFN-γ.

### Confocal microscopy analysis

HEK293KbC2 cells seeded on glass coverslips were transfected with the EBNA1-SIIN-GFP expression constructs as described above. At twenty-four hours post-transfection the cells were fixed in 4% paraformaldehyde for 20 mins, washed, permeabilized in 1% Triton-X100 in PBS for 20 mins, washed and then mounted in Pro Long Gold antifade reagent with DAPI (Molecular Probes, Invitrogen). GFP fluorescence in cells was detected using a laser-scanning Bio-Rad (Hercules, CA) MRC600 confocal microscope with original magnification ×63.

## Supporting Information

Text S1 Supporting Information including Figure S1 which shows mRNA sequences of the relevant repeat regions of the EBNA1 frameshift constructs and Figure S2 which shows the protein and mRNA sequences of the EBNA1 repeat region substituted with the *Ateline herpesvirus 3* repeat region.
(DOC)
