# Supplementary material for: Messenger RNA Sequence Rather than Protein Sequence Determines the Level of Self-synthesis and Antigen Presentation of the EBV-encoded Antigen, EBNA1
Source: PLoS Pathog. 2012 Dec 27;8(12):e1003112. doi: 10.1371/journal.ppat.1003112 (PMC3531512; doi:10.1371/journal.ppat.1003112)
Supplement: Text S1 — Supporting Information including Figure S1 which shows mRNA sequences of the relevant repeat regions of the EBNA1 frameshift constructs and Figure S2 which shows the protein and mRNA sequences of the EBNA1 repeat region substituted with the Ateline herpesvirus 3 repeat region. (DOC) [file ppat.1003112.s001.doc]

**Figure S1** mRNA sequences of the relevant repeat regions of the EBNA1 frameshift constructs used in this study. EBNA1 wild-type, frameshift 1 and frameshift 2 sequences are shown, indicating the start of the alternative reading frames resulting from one or two nucleotide deletions at the beginning of the repeat sequence and the return to native EBNA1 sequence by one or two nucleotide insertions at the end of the repeat sequence, respectively. A 611 bp DNA fragment (highlighted in yellow) containing 546 nucleotides of the EBNA1 native repeat sequence was cloned into the BspE*1* and mutated Cla*1* sites (highlighted in green) in the above sequences. To generate the frameshift 1 construct (GQE) a single (A) nucleotide (highlighted in red) was deleted at position 264 of the EBNA1 sequence. A (G) nucleotide was then inserted at EBNA1 position 809 (in blue) to maintain the wild-type EBNA1 reading frame immediately following the internal repeat. Similarly, to generate the frameshift 2 construct (GRS) two (A) nucleotides (highlighted in red) were deleted at position 264-265 of the EBNA1 sequence. Two nucleotides (AG) were inserted into the EBNA1 sequence at positions 813-814 (in blue) to maintain the wild-type EBNA1 reading frame following the internal repeat. The flanking nucleotides at the deletion sites in the frameshift 1 and frameshift 2 sequences are bolded and underlined.

E1-GA(wild-type)

atgtctgacgaggggccaggtacaggacctggaaatggcctaggagagaa

gggagacacatctggaccagaaggctccggcggcagtggacctcaaagaa

gagggggtgataaccatggacgaggacggggaagaggacgaggacgagga

ggcggaagaccaggagccccgggcggctcaggatcagggccaagacatag

agatggtgtccggagaccccaaaaacgtccaagttgcattggctgcaa

agggacccacggtggaacaggagcaggagcaggagcgggaggggcagg

agcaggaggggcaggagcaggaggaggggcaggagcaggaggaggggc

aggaggggcaggaggggcaggaggggcaggagcaggaggaggggcagg

agcaggaggaggggcaggaggggcaggaggggcaggagcaggaggagg

ggcaggagcaggaggaggggcaggaggggcaggagcaggaggaggggc

aggaggggcaggaggggcaggagcaggaggaggggcaggagcaggagg

aggggcaggaggggcaggagcaggaggaggggcaggaggggcaggagg

ggcaggagcaggaggaggggcaggagcaggaggggcaggaggggcagg

aggggcaggagcaggaggggcaggagcaggaggaggggcaggaggggc

aggaggggcaggagcaggaggggcaggagcaggaggggcaggagcagg

aggggcaggagcaggaggggcaggaggggcaggagcaggaggggcagg

aggggcaggagcaggaggggcaggaggggcaggagcaatcgatggagg

tggaggccggggtcgaggaggcagtggaggccggggtcgaggaggtagtg

E1-GQE(frameshift 1)

atgtctgacgaggggccaggtacaggacctggaaatggcctaggagagaa

gggagacacatctggaccagaaggctccggcggcagtggacctcaaagaa

gagggggtgataaccatggacgaggacggggaagaggacgaggacgagga

ggcggaagaccaggagccccgggcggctcaggatcagggccaagacatag

agatggtgtccggagaccccaaaaacgtccaagttgcattggctgcaa

agggacccacggtg**ga**caggagcaggagcaggagcgggaggggcagga

gcaggaggggcaggagcaggaggaggggcaggagcaggaggaggggca

ggaggggcaggaggggcaggaggggcaggagcaggaggaggggcagga

gcaggaggaggggcaggaggggcaggaggggcaggagcaggaggaggg

gcaggagcaggaggaggggcaggaggggcaggagcaggaggaggggca

ggaggggcaggaggggcaggagcaggaggaggggcaggagcaggagga

ggggcaggaggggcaggagcaggaggaggggcaggaggggcaggaggg

gcaggagcaggaggaggggcaggagcaggaggggcaggaggggcagga

ggggcaggagcaggaggggcaggagcaggaggaggggcaggaggggca

ggaggggcaggagcaggaggggcaggagcaggaggggcaggagcagga

ggggcaggagcaggaggggcaggaggggcaggagcaggaggggcagga

ggggcaggagcaggaggggcaggaggggcagGgagcaatcgatggagg

tggaggccggggtcgaggaggcagtggaggccggggtcgaggaggtagtg

E1-GRS(frameshift 2)

atgtctgacgaggggccaggtacaggacctggaaatggcctaggagagaa

gggagacacatctggaccagaaggctccggcggcagtggacctcaaagaa

gagggggtgataaccatggacgaggacggggaagaggacgaggacgagga

ggcggaagaccaggagccccgggcggctcaggatcagggccaagacatag

agatggtgtccggagaccccaaaaacgtccaagttgcattggctgcaa

agggacccacggtg**gc**aggagcaggagcaggagcgggaggggcaggag

caggaggggcaggagcaggaggaggggcaggagcaggaggaggggcag

gaggggcaggaggggcaggaggggcaggagcaggaggaggggcaggag

caggaggaggggcaggaggggcaggaggggcaggagcaggaggagggg

caggagcaggaggaggggcaggaggggcaggagcaggaggaggggcag

gaggggcaggaggggcaggagcaggaggaggggcaggagcaggaggag

gggcaggaggggcaggagcaggaggaggggcaggaggggcaggagggg

caggagcaggaggaggggcaggagcaggaggggcaggaggggcaggag

gggcaggagcaggaggggcaggagcaggaggaggggcaggaggggcag

gaggggcaggagcaggaggggcaggagcaggaggggcaggagcaggag

gggcaggagcaggaggggcaggaggggcaggagcaggaggggcaggag

gggcaggagcaggaggggcaggaggggcaggagcAGaatcgatggagg

tggaggccggggtcgaggaggcagtggaggccggggtcgaggaggtagtg

**Figure S2** Protein and mRNA sequences of the EBNA1 repeat region substituted with the *Ateline herpesvirus 3* repeat region. The 519 nucleotide purine-rich repetitive sequence (highlighted in yellow) of *Ateline herpesvirus 3* - ateles strain 73 ORF73 (AAC95598) was synthesized and cloned into the E1∆GA-SIIN-GFP expression vector to generate (E1-Ateline-SIIN-GFP).

Amino Acid sequence

MSDEGPGTGPGNGLGEKGDTSGPEGSGGSGPQRRGGDNHGRGRGRGRGRG

GGRPGAPGGSGSGPRHRDGVRRPQKRPSCIGCKGTHGGTGGDGRDGRDGR

DGRDGGDGGDGGDGGDGGDGEDGGDGGDGGDGGDGEEGGDGGDWEEGGDG

GDEGDEGDGGDGGDGGDGGDGEDGDDEDDGGDGGDGGDGGDGGDGEDGDD

EGDGGDGEDEDDGGDGGDGEDGGDGGDGGDGGDGGDGEDGEDGGDGGDGG

DGGDGGDGGEIDGGGGRGRGGSGGRGRGGSGGRGRGGSGGRRGRGRERAR

GGSRERARGRGRGRGEKRPRSPSSQSSSSGSPPRRPPPGRRPFFHPVGEA

DYFEYHQEGGPDGEPDVPPGAIEQGPADDPGEGPSTGPRGQGDGGRRKKG

GWFGKHRGQGGSNPKFENIAEGLRALLARSHVERTTDEGTWVAGVFVYGG

SKTSLYNLRRGTALAIPQCRLTPLSRLPFGMAPGPGPQPGPLRESIVCYF

MVFLQTHIFAEVLKDAIKDLVMTKPAPTCNIRVTVCSFDDGVDLPPWFPP

MVEGAAAEGDDGDDGDEGGDGDEGEEGQE*

mRNA sequence

atgtctgacgaggggccaggtacaggacctggaaatggcctaggagagaa

gggagacacatctggaccagaaggctccggcggcagtggacctcaaagaa

gagggggtgataaccatggacgaggacggggaagaggacgaggacgagga

ggcggaagaccaggagccccgggcggctcaggatcagggccaagacatag

agatggtgtccggagaccccaaaaacgtccaagttgcattggctgcaaag

ggacccacggtggaacaggaggagacgggagagacgggagagacgggaga

gacgggagagacgggggagacgggggagacgggggagacgggggagacgg

gggcgacggggaagacgggggcgatgggggagacggaggagacgggggag

acggggaagaggggggcgacgggggagactgggaagaggggggcgacggg

ggcgacgagggcgacgagggcgacgggggagacgggggagacgggggaga

cgggggagacggggaagacggggacgacgaggacgacgggggagacgggg

gagacgggggagacgggggagacgggggagacggggaagacggggacgac

gagggcgacgggggagacggggaagacgaggacgacgggggagacggggg

agacggggaagatgggggcgacgggggcgacgggggcgacgggggagacg

ggggcgacggggaagacggggaagacgggggagacgggggagacggggga

gacgggggagacgggggagacgggggagaaatcgatggaggtggaggccg

gggtcgaggaggcagtggaggccggggtcgaggaggtagtggaggccggg

gtcgaggaggtagtggaggccgccggggtagaggacgtgaaagagccagg

gggggaagtcgtgaaagagccagggggagaggtcgtggacgtggagaaaa

gaggcccaggagtcccagtagtcagtcatcatcatccgggtctccaccgc

gcaggccccctccaggtagaaggccatttttccaccctgtaggggaagcc

gattattttgaataccaccaagaaggtggcccagatggtgagcctgacgt

gcccccgggagcgatagagcagggccccgcagatgacccaggagaaggcc

caagcactggaccccggggtcagggtgatggaggcaggcgcaaaaaagga

gggtggtttggaaagcatcgtggtcaaggaggttccaacccgaaatttga

gaacattgcagaaggtttaagagctctcctggctaggagtcacgtagaaa

ggactaccgacgaaggaacttgggtcgccggtgtgttcgtatatggaggt

agtaagacctccctttacaacctaaggcgaggaactgcccttgctattcc

acaatgtcgtcttacaccattgagtcgtctcccctttggaatggcccctg

gacccggcccacaacctggcccgctaagggagtccattgtctgttatttc

atggtctttttacaaactcatatatttgctgaggttttgaaggatgcgat

taaggaccttgttatgacaaagcccgctcctacctgcaatatcagggtga

ctgtgtgcagctttgacgatggagtagatttgcctccctggtttccacct

atggtggaaggggctgccgcggagggtgatgacggagatgacggagatga

aggaggtgatggagatgagggtgaggaagggcaggagtga
